# ^99m^Tc-HYNIC-Annexin A5 in Oncology: Evaluating Efficacy of Anti-Cancer Therapies

**DOI:** 10.3390/cancers5020550

**Published:** 2013-05-15

**Authors:** Frédéric L.W.V.J. Schaper, Chris P. Reutelingsperger

**Affiliations:** Department of Biochemistry, Cardiovascular Research Institute Maastricht, MUMC, Universiteitssingel 50, 6200 MD Maastricht, The Netherlands

**Keywords:** ^99m^Tc-HYNIC-Annexin A5, annexine a5, molecular imaging, SPECT, cancer, efficacy of therapy

## Abstract

Evaluation of efficacy of anti-cancer therapy is currently performed by anatomical imaging (e.g., MRI, CT). Structural changes, if present, become apparent 1–2 months after start of therapy. Cancer patients thus bear the risk to receive an ineffective treatment, whilst clinical trials take a long time to prove therapy response. Both patient and pharmaceutical industry could therefore profit from an early assessment of efficacy of therapy. Diagnostic methods providing information on a functional level, rather than a structural, could present the solution. Recent technological advances in molecular imaging enable *in vivo* imaging of biological processes. Since most anti-cancer therapies combat tumors by inducing apoptosis, imaging of apoptosis could offer an early assessment of efficacy of therapy. This review focuses on principles of and clinical experience with molecular imaging of apoptosis using Annexin A5, a widely accepted marker for apoptosis detection *in vitro* and *in vivo* in animal models. ^99m^Tc-HYNIC-Annexin A5 in combination with SPECT has been probed in clinical studies to assess efficacy of chemo- and radiotherapy within 1–4 days after start of therapy. Annexin A5-based functional imaging of apoptosis shows promise to offer a personalized medicine approach, now primarily used in genome-based medicine, applicable to all cancer patients.

## 1. Introduction

The time span of a cancer drug entering a clinical trial until approval is approximately 8 to 10 years and therefore time consuming and costly. Primary and surrogate endpoints that indicate therapeutic efficacy such as overall survival (OS) and tumor response rates (TRR), respectively, necessitate an observation period of months to even years. In today’s standard clinical practice, TTR is regarded as the gold standard for evaluation of therapeutic effect and is widely used in oncologic clinical trials [1,2,3]. TRR focusses on the volumetric and morphometric assessment of lesions by means of anatomical imaging, e.g., magnetic resonance imaging (MRI), computed tomography (CT), X-ray or ultrasound. Evaluation of TRR is based on the Response Evaluation Criteria In Solid Tumors (RECIST) criteria [4]. TRRs are summarized in Table 1. Since TRR visualizes the direct effect of a drug on tumor size and regression of tumors in the absence of treatment is rare, TRR is attributable to treatment effect and for that reason a valid surrogate marker for efficacy of anti-cancer drugs. However, conventional anatomical imaging has several major drawbacks. Morphological changes first become apparent 1–2 months after start of therapy [5], which means that switching from a non-effective therapy to an effective therapy could take months. Patients could thus be exposed to unnecessary side effects and possibly be subject to disease progression. Also, volume measurements can be hindered by necrotic tissue and scar formation [6] and have the potential to be confounded by second line treatments [7]. 

**Table 1:** Summary of TRR and RECIST criteria as defined by Eisenhauer, Therasse *et al.* 2009 [4].

Tumor response rate	Abbreviation	RECIST
Complete response	CR	Disappearance of all target lesions
Partial response	PR	≥30% Decrease in the sum of diameters of target lesions
Progressive disease	PD	≥20% Increase in the sum of diameters of target lesions and an absolute increase of 5 mm
Stable disease	SD	Small changes that do not meet above criteria

Since most cancer therapies are aimed at inducing death of tumor cells [8], visualization of this toxic effect at the molecular level could provide a fast and direct method to assess anti-cancer drug efficacy. Molecular imaging (MI) could provide the solution. MI is a fusion between molecular biology and *in vivo* imaging that can visualize cellular processes. Advances in MI are expected to have a major impact on cancer detection, individualized treatment, drug development and understanding of how cancer arises [9]. The most significant advantage of MI compared to conventional imaging is that it offers disease information on a functional level as opposed to an anatomical level. Cancer, as any disease, is a pathologic biological process. Drugs are designed to interfere with the pathologic process and should thus also be validated using a functional screening method directed at these processes. Especially with the growing knowledge of the molecular players in cancer, the shift to personalized medicine and the possibility of theranostics in oncology, the need for a functional marker that can visualize disease processes and quantitate changes over time in a non-invasive nature, rises [10]. In MI, the target is the biological process, which is marked with a ligand that can be quantified. Because most cancer therapies combat tumors by inducing apoptosis and chemotherapy-induced apoptosis increases and peaks between 10 and 24 h after start of treatment [11,12,13,14], the biological process that could offer an early assessment (within 24 h) of efficacy of therapy is apoptosis.

## 2. Apoptosis

Apoptosis is a well-organized form of cell death that leads to the removal of cells from tissues without causing an inflammatory response. Apoptosis plays an essential role in programmed cell death (PCD) of early human development and in adult homeostasis, but is also a key feature of many forms of disease [15]. Apoptosis can be pathologic by both an excess and a lack of cell death. For instance in an acute myocardial infarct (AMI) occlusion of a coronary artery causes ischemia, depriving the myocard of oxygen and nutrients, and stresses the heart. When blood flow is reestablished (reperfusion) the infarcted myocard gets flooded by a pool of inflammatory signals and cells, causing a heart tissue targeted immune response. The ischemic stress factor and reperfusion injury cause both an inflammatory form of cell death (necrosis) and a non-inflammatory form of cell death (apoptosis). Evidence suggests that apoptosis plays a major role in the tissue damage caused by ischemia/reperfusion (I/R) injury in AMI patients [16,17]. Though in AMI and neurodegenerative disorders such as Alzheimer’s disease there is an excess of cell death, in cancer there is a lack. Cancer is caused by too much proliferation and/or too little degeneration. Derailment of apoptosis could thus create a state in which cell proliferation exceeds cell death, thereby producing a tumor [18]. Induction of apoptosis could thus also contribute to the regression of tumors. Hence, in depth knowledge on the molecular mechanisms governing apoptosis will provide rationale not only to novel therapeutic avenues but also to diagnostic strategies to evaluate early response to therapy.

The molecular mechanisms of apoptosis have been described in detail elsewhere [19]. In short, there are two main apoptotic signaling cascades: the extrinsic and intrinsic pathway. The extrinsic pathway is activated by receptor binding of death ligands (e.g., tumor necrosis factor or Fas ligand) and the intrinsic pathway is activated by internal cellular stressors (e.g., DNA damage or chemotherapeutic agents). Though the proteins involved in controlling and driving the apoptotic machinery differ, both pathways ultimately lead to the activation of the major effector caspases 3, 6 and 7. Subsequently, key cellular structures and organelles are demolished and various structural and membrane changes are initiated that characterize the apoptotic cell phenotype. Phagocytes recognize this phenotype by particular membrane changes and engulf them to complete cell suicide, clearing them from the tissue. These suicide signals, or “eat me” flags, are membrane bound molecules that interact with receptors on the phagocyte. Though there are many different “eat me” flags and their different contributions to phagocyte attraction are still unclear, one of the essential membrane changes in an apoptotic cell has been determined to be the externalization of phosphatidylserine (PS) [20].

## 3. Phosphatidylserine Externalization

In 1992, Fadok *et al.* [21] discovered that apoptotic cells expose PS on the outer leaflet of the plasma membrane (PM). In viable cells, PS is only present in membrane leaflets facing the cytosol. When cells become apoptotic, regardless of the initiating stimulus, the membrane phospholipid PS is exteriorized making it visible for phagocytes [22]. Phagocytes have a specific PS receptor to recognize apoptotic cells and engulf them [23]. Before PS is externalized, the cell must have activated a series of biochemical reactions to initiate apoptosis. One can distinguish between an early apoptotic cell characterized by PS externalization, caspase activation, DNA fragmentation and chromatin condensation, and a late apoptotic cell characterized by cell shrinkage, membrane blebbing, cell fragmentation and apoptotic body formation [24]. PS externalization is thus an event occurring in early apoptosis and, as described earlier, signals phagocytes to recognize and engulf the apoptotic bodies without causing an inflammatory response [25,26]. Though PS exposure on the outer leaflet of the cell membrane has been a well-studied death phenotype, the molecular link between apoptosis and PS externalization is still unclear [19]. Detailed information on the intracellular changes and proteins involved so far are described elsewhere [27,28,29,30,31].

Externalization of PS is not restricted to apoptosis but has been established in activated platelets, aging erythrocytes, activated endothelium of tumor vasculature, activated macrophages, megakaryocytes, necrosis and autophagy [32,33]. Interestingly, stressed cells have also shown to expose PS on their PM [34], but not when the stressor is removed. This reversible form of PS externalization is characterized by lower levels of PS exposure compared to apoptotic cells. PS exposure can thus not only be a sign for cells undergoing apoptosis, but also for (temporary) stressed cells with a high risk to become apoptotic [26]. Although PS externalization can be present in a variety of cells, apoptosis is regarded as the most important and abundant cellular process accompanied by the PS death phenotype. 

## 4. Annexin A5

Since an excess or lack of apoptosis are key factors of disease, detection of apoptosis could contribute to localize pathological sites, study disease progression, support diagnosis and assess efficacy of therapy [35]. The discovery of PS externalization has made way for finding compounds that have an affinity for PS and could therefore be used to study apoptotic sites. To date, one compound has received major interest in both the preclinical and clinical arena: Annexin A5.

Annexin A5 is a non-glycosylated single chain protein physiologically involved in inhibition of hemostasis. It is part of a protein family that binds to negatively charged phospholipids in a Ca^2+^-dependent manner. The ligand Annexin A5, used for research purposes, is produced by the expression of Annexin A5 complementary DNA in *Escherichia coli*. Though Annexin A5 is not the only PS binding compound (others include: C2A domain of synaptotagmin 1, lactadherin, T cell immunoglobin mucins, γ-carboxyglutamic acid (Gla) containing proteins, PS antibodies), it has several advantages. Annexin A5 possesses a Kd of 0.1–2 nM, which constitutes in a high PS binding [26,30,31,35]. Furthermore, a PS expressing cell can internalize Annexin A5, opening possibilities for targeted drug delivery (TDD) [36]. Nevertheless, the greatest advantage of Annexin A5 with regard to clinical implementation is the wide preclinical and clinical experience in the use of this compound. In translational research, Annexin A5 is used in an apoptosis detection assay [37,38] in conjunction with propidium iodide to distinguish between apoptotic and necrotic cells, but is also labeled with radionuclides for measuring apoptosis *in vitro* and *in vivo* in animal models and patients [16,39,40]. Since PS is a key factor in the phagocytic clearance of dying cells, it has been hypothesized that binding of Annexin A5 could inhibit this process and therefore interfere with the inflammatory and immunologic responses to the dying cell [41,42]. Indeed it has been found that Annexin A5 can inhibit phagocytosis by internalizing the PS-expressing membrane patch [32,43]. However, it does so without interfering with the key steps of the apoptotic program [36]. It has been reported that binding of Annexin A5 to PS can accelerate [44], but also delay [45] the apoptotic cell death program. The influence of Annexin A5 on the cell death program is thus not clear but seems to be dependent on cell type and cell death trigger [32]. 

## 5. Annexin A5 Imaging of Cell Death

*In vivo* imaging of apoptosis by means of Annexin A5 can only become clinically relevant if there is a quantitative, repetitive and non-invasive way to measure Annexin A5 tissue uptake. Several radiopharmaceutical probes have been designed to image Annexin A5 uptake by means of SPECT, PET, MRI and near-infrared fluorescence (NIRF). Of these radioligands, only a few have been studied in humans, namely the SPECT probes: ^99m^Tc-HYNIC-Annexin A5, ^99m^Tc-EC-Annexin A5, ^99m^Tc-BTAP-Annexin A5 (also known as ^99m^Tc-Apomate) and ^123^I-Annexin A5 [24].

Technetium-^99m^ (^99m^Tc) is the most commonly used medical radioisotope and can be detected using imaging tools equipped with gamma cameras, such as SPECT. ^99m^Tc is routinely used in nuclear medicine in for instance bone scintigraphy, myocardial perfusion imaging and functional brain imaging. ^99m^Tc cannot be coupled directly to Annexin A5 but requires explicit conjugation principles. Various methods have been used in clinical practice so far employing amide bonds (hydrazinonicotinamide [HYNIC]), disulfur dinitride (N_2_S_2_) linking with ethylenedicysteine (EC) and 4,5-bis(thioacetamido)pentanoyl (BTAP) [39]. Thanks to the pioneering work of Belhocine *et al.*, using ^99m^Tc-BTAP-Annexin A5 to monitor chemosensitivity in a variety of cancer types (e.g., lung cancer, lymphoma and breast cancer), clinical studies investigating the various tracers described above followed [46,47,48,49,50]. Interestingly, ^99m^Tc-BTAP-Annexin A5 uptake 24–48 h after the first course of chemotherapy was significantly related to survival and progression-free survival in lung cancer and lymphoma patients [51]. This finding provides the first clinical evidence that Annexin A5 imaging could be used to assess efficacy of anti-cancer treatments 24 h after treatment. However, slow blood clearance, gut uptake and time consuming and elaborate preparation makes this tracer unsuitable for clinical practice [46,48,52]. The most widely applied tracer for clinical use of Annexin A5-based functional imaging is ^99m^Tc-HYNIC-Annexin A5 [24]. ^99m^Tc-HYNIC-Annexin A5 is available as a good manufacturing practice (GMP) product in a radiolabeling kit. A phase 1 [46] study showed that highest uptake of tracer was observed in the kidneys (30 min and 24 h after injection), followed by the liver and spleen, but no uptake in the gut 24 h post injection. Blood pool activity was cleared for more than 90%, with a half-life of 24 min. ^99m^Tc-HYNIC-Annexin A5 allows for imaging at 4–6 h after tracer injection. ^99m^Tc-EC-Annexin A5 is an alternative candidate for apoptosis imaging, but compared to ^99m^Tc-HYNIC-Annexin A5 and ^99m^Tc-BTAP-Annexin A5, there is little experience [47]. In an attempt to reduce renal uptake, Annexin A5 was labeled with the halogen radioisotype ^123^I. ^123^I-Annexin A5 did indeed show good abdominal region imaging compared to ^99m^Tc compounds (no liver and renal tracer uptake 12 h post injection), but is subject to rapid *in vivo* dehalogenation, is more expensive and has a more complex labeling method [39,53]. Although most radiopharmaceuticals are designed for SPECT, PET has several advantages over SPECT. PET offers a higher resolution, higher sensitivity and more accurate quantification. Drawbacks of PET imaging are the higher costs and use of a cyclotron for the production of short-lived radionuclides. Two Annexin A5 PET radionuclides have shown promise, but have only been tested in the preclinical arena: ^18^F-Annexin A5 and ^68^Ga-Annexin A5. The great advantage of ^18^F-Annexin A5 is that it is a PET radionuclide with an optimal half-life for diagnostic imaging (110 min). However, currently labeling procedures are time-consuming and not yet realistic for a clinical setting. PET imaging with ^68^Ga-Annexin A5 could be more realistic, since it also possesses a short half-life (68 min), which allows for rapid imaging and labeling procedures are more straightforward. Unfortunately, there are no clinical studies describing the use of PET radionuclides to date, but are likely to become apparent soon [24]. Although SPECT and PET imaging of apoptosis are probably the most sensitive, they lack specificity due to their poor anatomic mapping. New imaging tools that combine molecular and anatomic imaging such as SPECT/CT, PET/CT and PET/MRI could offer best from both worlds [35] and have been of use in various clinical studies [54,55]. Although different radiopharmaceutical probes have been labeled to Annexin A5, to date, the best candidate to become widely applied in clinical practice in the near future is ^99m^Tc-HYNIC-Annexin A5. 

## 6. ^99m^Tc-HYNIC-Annexin A5 Evaluation of Efficacy of Anti-Cancer Therapies

For evaluation of efficacy of anti-cancer therapies, ^99m^Tc-HYNIC-Annexin A5 imaging has to be performed both before (baseline) and after start of treatment (ASOT). To assess the significance of ^99m^Tc-HYNIC-Annexin A5 tumor uptake at baseline, clinical studies investigating baseline measurements of homogenous populations of head and neck cancer (HNC) patients are described first.

Successful anti-cancer treatment is expected to have a high apoptosis inducing potential and should thus reflect in increased ^99m^Tc-HYNIC-Annexin A5 tumor uptake ASOT compared to baseline. Clinical studies investigating the evaluation of therapy response by means of ^99m^Tc-HYNIC-Annexin A5 imaging before and ASOT are described second. These studies comprise a heterogeneous population of HNC, lymphoma, non-small cell lung cancer (NSCLC), breast cancer (BrC), melanoma and various other cancer types. Therapeutic interventions (e.g., chemo- and/or radiation-therapy) varied. 

A summary of all clinical studies of ^99m^Tc-HYNIC-Annexin A5 imaging in oncology is provided in Table 2. 

### 6.1. Baseline Measurements

The first to report the use of ^99m^Tc-HYNIC-Annexin A5 in oncology patients were Van de Wiele *et al.* in 2003 [56]. They studied the relation between baseline tumor tissue uptake of ^99m^Tc-HYNIC-Annexin A5 and apoptosis. 18 HNC patients underwent a CT scan for anatomical imaging of tumor dimensions and a ^99m^Tc-HYNIC-Annexin A5 SPECT scan for apoptosis imaging. Tumors were surgically resected within 10 days after SPECT imaging and apoptotic cells were histopathologically quantified using terminal deoxynucleotidyl transferase-mediated deoxyuridine triphosphate-biotin nick end-labeling (TUNEL) assays. Necrotic cells were estimated semiquantatively by means of hematoxylin and eosin (HE) staining. The authors found that ^99m^Tc-HYNIC-Annexin A5 tumor uptake 5 to 6 h post injection (PI) correlates statistically significant with the number of apoptotic cells found by TUNEL assays when samples with no or minimal amounts of necrosis are considered. Increasing amounts of necrosis resulted in a progressive decrease of correlation. ^99m^Tc-HYNIC-Annexin A5 tumor uptake 5 to 6 h PI is thus a good reflection of ongoing apoptosis *in vivo*, but only in tissues with no or minimal necrosis. These data indicate that necrosis can reduce the apoptosis imaging potential of ^99m^Tc-HYNIC-Annexin A5. ^99m^Tc-HYNIC-Annexin A5 binding to PS on the inner leaflet of the cell membrane could explain this, since the PM is well known to be disrupted in necrosis. Preclinical and clinical data of ^99m^Tc-HYNIC-Annexin A5 imaging in myocardial infarction support this [16,22,57]. 

In 2004, the same group published a paper [58] estimating the intra-, inter-, and day-to-day reproducibility of manually defined quantitative ^99m^Tc-HYNIC-Annexin A5 tumor uptake values in 11 HNC patients. Vermeersch *et al.* state that “for clinical application, sufficient reproducibility must be demonstrated to allow for a study of cell-death changes induced by chemotherapy over time and intersubject”. They found a mean −3.4%, 2.4%, and −6% difference for the intra-, inter-, and day to day measurements, respectively. No systemic bias was observed. The authors conclude that “the reproducibility of quantitative ^99m^Tc-HYNIC-Annexin A5 tumor uptake measurement using a manual method appears to be acceptable for clinical use”.

That same year, Vermeersch *et al.* [59] describe 18 HNC patients who underwent baseline ^99m^Tc-HYNIC-Annexin A5 imaging followed by surgical resection and lymph node dissection. 5 to 6 h PI, ^99m^Tc-HYNIC-Annexin A5 was taken up in all primary HNC lesions identified by CT, but in 5 of the 6 patients there was no tracer uptake in involved lymph nodes as opposed to CT. “^99m^Tc-HYNIC-Annexin A5 thus allowed for the visualization of all primary HNC tumors identified by CT scan, but failed to identify most of the sites of lymph node involvement”. The authors attribute this to the low resolution of the gamma camera and the small lymph node sizes (<15 mm). Tracer biodistribution entails the bone marrow, liver, bladder and kidneys with no blood pool activity 5 to 6 h PI. Delineation of small lesions close to these structures is thus unlikely when using ^99m^Tc-HYNIC-Annexin A5. 

In 2007, the group [60] reports pre-treatment ^99m^Tc-HYNIC-Annexin A5 SPECT imaging in 23 patients of various cancer types. Patients received radio- and/or chemotherapy and TRR were defined by RECIST criteria 2–3 and 5–6 months ASOT. ^99m^Tc-HYNIC-Annexin A5 tumor-to-background ratio (T/N) was found to be significantly higher in responders compared to non-responders. However, due to the heterogeneous nature of the group of patients and possible difference in presence of necrosis, no single T/N threshold could be determined to distinguish between responders and non-responders. 

One year later the same group reports [61] the possibility of baseline ^99m^Tc-HYNIC-Annexin A5 T/N to be of prognostic value in HNC. They refer to several papers [62,63,64,65] that describe a high apoptotic index of tumor, as defined by hispathological analysis, to be associated with a poor overall survival. 29 HNC patients underwent ^99m^Tc-HYNIC-Annexin A5 SPECT scans at baseline (4 to 6 h PI), followed by surgical resection, lymph node dissection and/or (chemo)radiation therapy. Median follow up was 22.6 months. Biodistribution was similar to previous studies. They found that ^99m^Tc-HYNIC-Annexin A5 T/N was inversely correlated with disease-free survival (r = −0.684, *p* = 0.000) and overall survival (r = −0.669, *p* = 0.000). Because no histopathological analysis was performed, the authors remark that part of the image signal obtained, may be attributable to necrosis rather than apoptosis. On contrast enhanced CT however, necrotic sites were only seen in two patients. To validate the prognostic value of ^99m^Tc-HYNIC-Annexin A5 imaging in HNC, the authors state that a bigger sample size is needed. 

**Table 2:** Clinical studies of ^99m^Tc-HYNIC-Annexin A5 in oncology.

Reference	Patients (n)	Imaging time-points	Aim of the study	End points	Results
Van de Wiele *et al.* 2003 [56]	HNC (18)	Baseline	Identifying the relationship between baseline quantitative ^99m^Tc-HYNIC-Annexin A5 tumor uptake and the number of apoptotic cells derived from histologic analysis after surgical resection.	n.a.	Quantitative ^99m^Tc-HYNIC-Annexin A5 tumor uptake correlated well with the number of apoptotic cells if only tumor samples with no or minimal amounts of necrosis were considered.
Vermeersch, Ham *et al.* 2004 [58]	HNC (11)	Baseline	Estimation of the intra-, inter-, and day-to-day reproducibility of quantitative ^99m^Tc-HYNIC-Annexin A5 tumor uptake values.	n.a.	The mean differences for the intra-, inter-. and day-to-day measurements were −3.4%, 2.4%, and −6%, respectively.
Vermeersch, Loose *et al.* 2004 [59]	HNC (18)	Baseline	^99m^Tc-HYNIC-Annexin A5 visualization of primary HNC lesions and lymph nodes before surgical resection and lymph node dissection.	n.a.	^99m^Tc-HYNIC-Annexin A5 allowed for the visualization of all primary HNC tumors identified by CT scan, but failed to identify most of the sites of lymph node involvement.
Haas *et al.* 2004 [66]	FL (11)	Baseline + up to 48 h ASOT	Evaluation of ^99m^Tc-HYNIC-Annexin A5 imaging for monitoring radiation-induced apoptotic cell death.	n.a.	In 10 patients, post-treatment cytology matched ^99m^Tc-HYNIC-Annexin A5 uptake ASOT. Baseline uptake was weak or absent.
Kartachova *et al.* 2004 [67]	FL (22) NSCLC (5) HNC (2)	Baseline + up to 72 h ASOT	Predicting outcome of various treatments by ^99m^Tc-HYNIC-Annexin A5 imaging.	TRR	Only patients with a CR or PR showed a significant increase in ^99m^Tc-HYNIC-Annexin A5 uptake ASOT.
Rottey *et al.* 2006 [71]	M (3) Bl (1) BrC (5) HNC (2) Other (6)	Baseline + 5–7 and 40–44 h ASOT	Predicting outcome of chemotherapy by ^99m^Tc-HYNIC-Annexin A5 imaging.	TRR	^99m^Tc-HYNIC-Annexin A5 imaging allowed for separation of responders and non-responders to treatment in 16 of the 17 patients.
Rottey *et al.* 2007 [60]	HNC (8) BrC (6) M (2) Other (7)	Baseline	Predicting outcome of (radio)chemotherapy by baseline uptake of ^99m^Tc-HYNIC-Annexin.	TRR	Significantly higher pre-treatment tracer uptake was found in therapy responders (CR, PR) compared to non-responders (PD, SD).
Kartachova *et al.* 2007 [68]	NSCLC (14)	Baseline + up to 48 h ASOT	Predicting outcome of platinum-based chemotherapy by ^99m^Tc-HYNIC-Annexin A5 imaging.	TRR	Patients with notably increased ^99m^Tc-HYNIC-Annexin A5 uptake showed CR or PR. SD or PD showed less prominently increased or decreased tracer uptake.
Kartachova *et al.* 2008 [69]	NSCLC (4) HNC (3) FL (26)	Baseline + 24–48 h ASOT	Identifying the reliability of visual analysis of ^99m^Tc-HYNIC-Annexin A5 tumor uptake compared to quantitative tracer uptake evaluation.	TRR	Both visual (r = 0.97, *p* < 0.0001) and quantitative (r = 0.99, *p* < 0.0001) analysis of ^99m^Tc-HYNIC-Annexin A5 tumor uptake significantly correlated with TRR.
Hoebers *et al.* 2008 [70]	HNC (13)	Baseline + up to 24 h ASOT	Predicting outcome of cisplatin-based chemoradiation by ^99m^Tc-HYNIC-Annexin A5 imaging.	TRR DFS OS	^99m^Tc-HYNIC-Annexin A5 imaging showed a radiation-dose-dependent uptake in parotid glands. No correlation could be established between baseline or treatment induced tracer uptake and TRR, DFS or OS.
Loose *et al.* 2008 [61]	HNC (29)	Baseline	Identifying prognostic value of baseline ^99m^Tc-HYNIC-Annexin A5 imaging.	DFS OS	^99m^Tc-HYNIC-Annexin A5 pre-treatment uptake was inversely correlated with DFS and OS.
Rottey *et al.* 2009 [72]	HNC (4) BrC (2) Other (5)	2× Baseline within 40–44 h from each other or baseline + 5–7 and 40–44 h ASOT	Determining the influence of chemotherapy on the biodistribution of ^99m^Tc-HYNIC-Annexin in healthy tissues.	n.a.	No significant differences in ^99m^Tc-HYNIC-Annexin uptake in healthy tissues were found between patients which received chemotherapy and which did not.

HNC, head and neck cancer; CT, computed tomography; DFS, disease free survival; OS, overall survival; TRR, tumor response rate; ASOT, after start of therapy; CR, complete response; PR, partial response; SD, stable disease; PD, progressive disease; FL, follicular lymphoma; NSCLC, non-small cell lung cancer; M, melanoma; Bl, bladder; BrC, breast cancer; n.a., not applicable.

### 6.2. Therapy Response Measurements

In 2008, Verheij provides us with an excellent review [55] of their work with ^99m^Tc-HYNIC-Annexin A5 imaging of therapy induced cell death. The review entails the studies performed by Haas *et al.* [66] and Kartachova *et al.* [67,68]. Haas *et al.* performed ^99m^Tc-HYNIC-Annexin A5 imaging (4 h PI) in 11 low grade follicular lymphoma (FL) patients before and up to 48 h after radiotherapy. In six patients baseline tracer uptake was absent and in five patients weak. ^99m^Tc-HYNIC-Annexin A5 uptake ASOT matched the post-treatment cytology of apoptotic cell death (defined by fine needle aspiration) in 10 patients. Kartachova *et al.* describe FL, NSCLC and HNC patients (n = 29), which underwent ^99m^Tc-HYNIC-Annexin A5 imaging (4 h PI) before and up to 72 h after various treatments (radiotherapy, platinum-based chemotherapy or chemoradiation) [67]. Tumor response to therapy was assessed using ultrasonography, CT and/or MRI and defined by RECIST criteria 1–3 months ASOT. Patients with a CR or PR showed a significant increase in ^99m^Tc-HYNIC-Annexin A5 tumor uptake ASOT. Patients with SD or PD showed absent or low tracer uptake before therapy with no significant increase ASOT. Kartachova *et al.* showed similar results of ^99m^Tc-HYNIC-Annexin A5 imaging (4 h PI) before and up to 48 h after start of platinum-based chemotherapy in 14 NSCLC patients [68]. In his review of these studies, Verheij collected the raw data and correlated changes in ^99m^Tc-HYNIC-Annexin A5 uptake (∆U) with TRR. A highly significant correlation (r^2^ = 0.86, *p* < 0.0001) was found, indicating an increased tumor uptake of ^99m^Tc-HYNIC-Annexin A5 ASOT to be a potential predictor of clinical therapy outcome. 

In their previous reports, Verheij and co-workers quantified ^99m^Tc-HYNIC-Annexin A5 by visual analysis. ^99m^Tc-HYNIC-Annexin A5 uptake was expressed as a four grade score: 0 = absent, 1 = weak, 2 = moderate, 3 = intense. In 2008, Kartachova and co-workers [69] compared visual analysis with the current ‘gold standard’: quantitative uptake evaluation. Quantitative evaluation assesses the maximal counts per pixel in the tumor volume (C_max_) and was performed by an experienced operator, using a conventional nuclear medicine workstation. C_max_ changes were expressed as percentages of baseline values: grade −1, decrease >25%; grade 0, decrease between 1 and 25%; grade 1, 1–25% increase; and grade 2, >25% increase. In 79% of patients, visual and quantitative analysis agreed on tracer uptake. When in disagreement (n = 6), visual analysis underscored tracer uptake in five of the six patients. Both visual (r = 0.97, *p* < 0.0001) and quantitative (r = 0.99, *p* < 0.0001) analysis of ^99m^Tc-HYNIC-Annexin A5 tumor uptake correlated significant with TRR. The authors conclude that visual evaluation of ^99m^Tc-HYNIC-Annexin A5 tumor uptake appears to be a reliable method to detect early treatment-induced apoptosis and predict tumor response. A study of the same group published in 2008 [70], showed the opposite. Hoebers and co-workers reported a radiation-dose-dependent uptake in parotid glands of 13 HNC patients, but no correlation could be established between baseline or treatment induced tracer uptake and TRR, DFS or OS. The authors attribute this to the possible presence of necrosis in advanced stages of HNC, lymphocyte infiltration and small sample size.

In 2006, Rottey and colleagues [71] studied the changes in ^99m^Tc-HYNIC-Annexin A5 tumor uptake after chemotherapy in a variety of patients. Seventeen patients received tracer injections before and 5–7 and 40–44 h ASOT. T/N was calculated and TRR were defined by RECIST criteria 3 and 6 months ASOT. A 25% increase in T/N compared to baseline was considered significant. ^99m^Tc-HYNIC-Annexin A5 imaging in conjunction with the 25% threshold for significance allowed for a 94% (16/17 patients) accuracy of separation of responders and non-responders. The sensitivity, specificity, positive predictive value and negative predictive value were 86%, 100%, 100%, 91%, respectively. 

Three years later, Rottey and co-workers [72] investigated the biodistribution of ^99m^Tc-HYNIC-Annexin A5 in healthy tissues (liver, kidney, spleen, bone marrow, total body). To determine the influence of a previous dose of ^99m^Tc-HYNIC-Annexin A5, two scans within 40–44 h from each other were performed. Patients (n = 5) did not receive any treatment between the two tracer injections. No significant differences between the two scans were found for any healthy human tissue. To determine the influence of administration of chemotherapy, patients (n = 6) underwent pretreatment, 5–7 h and 40–44 h post treatment scans. No significant differences were found between the three scans. The authors conclude that neither a previous injection of ^99m^Tc-HYNIC-Annexin A5, nor administration of chemotherapy interferes with tracer uptake in healthy human tissues. 

As described above, various studies performed during the past decennium have indicated that ^99m^Tc-HYNIC-Annexin A5 could be capable of predicting therapy response at baseline and ASOT, can be easily quantified and is not hampered by cumulative tracer injections or chemotherapy. ^99m^Tc-HYNIC-Annexin A5 imaging could therefore possibly identify those patients who will benefit from the anti-cancer therapy at an early stage (within 24–48 h ASOT). 

## 7. Implications for Health Care

Both the pharmaceutical industry and patient could benefit from Annexin A5 imaging. Current evaluation of efficacy of anti-cancer therapy is performed by conventional anatomical imaging, which may take 1–2 months [5] to show first signs of tumor shrinkage. This culminates into lengthy clinical trials and a risk for cancer patients to receive an ineffective therapy and suffer from unnecessary side effects. Since Annexin A5 tumor uptake has shown promise to be a predictor of TRR, DFS and OS, Annexin A5 imaging could be used as a surrogate endpoint in clinical trials, subsequently lead to faster drug approval and even lower the economic burden on pharmaceutical industries. Molecular imaging of Annexin A5 in the drug development stage could accelerate the search for novel anti-cancer strategies. In oncology practice, the earlier recognition of treatment (in)efficacy should allow for faster clinical decision making, reduction of unnecessary side effects, shorten treatment period and improve quality of life of the patient [73]. Moreover, since the treatment plan is adjusted to the therapy response of the individual patient, Annexin A5 imaging is in line with recent advances in personalized medicine. Accordingly, when used for every cancer patient receiving their first course of chemo- or radiotherapy, Annexin A5 apoptosis imaging could introduce itself as a general diagnostic method to provide personalized medicine. 

Although Annexin A5 imaging offers a personalized medicine centered approach without collecting genetic information, discrimination by insurers could become apparent when implemented in clinical practice. The ethical problem that could arise is described as follows. If cancer treatment is financed by an insurer, this is because it has shown beneficial effects on clinical trial end points in a vast statistical group. Accordingly, the reason for an insurer to finance treatment is the proven therapeutic effect in large populations. When using Annexin A5 imaging, the choice of therapy is not based on a statistical reference group, but on the individual patient. Treatment plans could thus not be covered by the insurer, because of discordance between the individual and population therapy response. For an insurer it seems illogic to finance an ineffective therapy, even if it is the first therapy of choice as defined by the statistical group. Functional imaging could thus influence which treatments are financed by insurers and which are not. Therefore, communication on regulations is indispensable before clinical implementation of Annexin A5 imaging. 

Before Annexin A5 imaging is to be used in clinical practice, one has to wonder whether expensive diagnostics are needed if standard treatment plans are relatively cheap and have a good probability of success. However, with the increasing amounts and costs of cancer medications, early assessment of efficacy of therapy by Annexin A5 imaging could offer an economic advantage. Nonetheless, extensive research is needed to validate Annexin A5 imaging and produce various radiopharmaceutical probes for different imaging strategies. One could question if this investment is worthwhile. Given that survival benefits of newly developed, often expensive, cancer drugs (e.g., biologicals) are expressed in months [74,75], early diagnostics by means of Annexin A5 could provide an economically favorable way of ‘buying time’ compared to high end treatment plans. 

## 8. Discussion

With the current shift towards personalized medicine in oncology and the unmet medical needs of conventional imaging, Annexin A5 based functional imaging could become part of standard clinical practice in the future. Before implementation, a few issues have to be discussed. 

*First: What is the significance of an Annexin A5 positive signal*? Several studies have shown that PS externalization is not restricted to apoptosis. Annexin A5 binding of PS has been found in a variety of cell death mechanisms (e.g., apoptosis, necrosis, autophagy), tumor vasculature, inflammation and activated platelets [73]. In preclinical research, the environment can be controlled and Annexin A5 in conjuction with propidium iodide, is used to differentiate between apoptosis and necrosis [38]. In the clinical situation however, this is not possible. Studies in patients with cardiovascular disease have shown that intracardiac uptake of Annexin A5 can be caused by myocardial infarction, ongoing heart failure, intracardiac tumor and/or an infection [76]. Annexin A5 imaging for diagnostics is thus not to be used solely, but as an addition to standard diagnostic methods. In oncology, the lack of apoptotic specificity of Annexin A5 could work in its advantage. Cancer therapy does not kill cancer cells only by induction of apoptosis, but by a variety of cell death signaling pathways [73]. Since PS exposure to the environment is not restricted to apoptosis, Annexin A5 could be used as a universal marker of cell death and study pathologic sites *in vivo* in a non-invasive way. 

*Second: Which radiopharmaceutical probes and imaging techniques should be used?* Although there are a variety of Annexin A5 probes for both SPECT, PET, MRI and even NIRF [24], for near future clinical implementation, experience is of most importance. Most clinical studies used ^99m^Tc-HYNIC-Annexin A5 and SPECT imaging. These studies showed that tracer uptake could be both of prognostic value and of use in evaluation of efficacy of anti-cancer therapies (Table 2). Biodistribution also seems favorable compared to other tracers [46]. At the present time, ^99m^Tc-HYNIC-Annexin A5 is thus the best candidate for clinical implementation. For the future, PET tracers are expected to be developed to profit from the high resolution of PET imaging. 

*Third: Which patients do we evaluate*? In theory, all cancer patients are eligible. However, due to biodistribution, malignancies of the kidneys, liver, spleen and bone marrow are expected to be overlooked. Advances in tracer development, using mutated “second generation” Annexin A5, could solve this problem [77,78]. To date, most experience of ^99m^Tc-HYNIC-Annexin A5 apoptosis imaging has been with HNC, FL, NSCLC and BrC patients. 

*Fourth: What are the current problems*? One problem is the high renal retention and biodistribution of ^99m^Tc-HYNIC-Annexin A5, blurring the scan in these regions. Improvements in distinguishing physiologic tracer uptake from pathologic, have been made by use of SPECT/CT fusion images [55]. Yet, optimization and standardization between studies is needed. The major problem of the clinical studies described, is the heterogeneous patient populations investigated. These studies entail patient populations of various cancer types receiving numerous kinds of treatments. Moreover, defined study end points are not always concordant. Accordingly, there is a need for more standardized clinical studies of homogeneous patient populations, using pre-defined end points such as TRR, DFS and OS. 

*Fifth: If so promising, why has Annexin A5 imaging not been widely applied?* Still, in spite of advances in tumor imaging, the gold standard of clinical trial endpoints remains TRR and OS. However, this culminates into a long duration and high costs of clinical trials. Accordingly, if to be of use for both the pharmaceutical industry and the individual patient, ^99m^Tc-HYNIC-Annexin A5 imaging must be able to accurately and reproducibly predict the outcome of phase 3 clinical trials (e.g., DFS and OS) in a short time window [79,80]. Only then, organizational and economic wins are anticipated. The implementation of ^99m^Tc-HYNIC-Annexin A5 into standard clinical practice has two requirements: scientific validation (quantitative, reproducible, specific, sensitive) and logistic feasibility [10]. Since, from an ethical perspective ^99m^Tc-HYNIC-Annexin A5 imaging is not substantially different from conventional diagnostic tools and Annexin A5 costs will decrease when more tracers hit the market and popularity grows, logistic feasibility is not expected to be a “deal-breaker”. Scientific validation, however, needs large patient populations and specialized physicians, is impeded by Food and Drug Administration (FDA) regulations, produces high costs and is time-consuming. Pharmaceutical and diagnostic industries will thus have to play a key role in the scientific validation of ^99m^Tc-HYNIC-Annexin A5 imaging in evaluating efficacy of anti-cancer therapies.

## 9. Conclusions

^99m^Tc-HYNIC-Annexin A5 imaging shows potency to predict efficacy of anti-cancer therapy and thereby bears the promise to assess therapy response in a personalized manner at an early stage in cancer treatment. More clinical studies are required to validate Annexin A5-based functional imaging as a surrogate endpoint before standard clinical implementation is to be expected. 
